# The Guardian of Dreams: The Neglected Relationship Between Sleep and Psychoanalysis

**DOI:** 10.3390/brainsci15030281

**Published:** 2025-03-06

**Authors:** Giuseppe Barbato

**Affiliations:** Department of Psychology, Università degli Studi della Campania, Luigi Vanvitelli, 81100 Caserta, Italy; giuseppe.barbato@unicampania.it

**Keywords:** dreaming, psychoanalysis, sleep, Freud, REM sleep, sleep disturbances

## Abstract

Knowledge about sleep was very limited at the time when Freud published his seminal work on the interpretation of dreams. He was also not interested in sleep, which was considered a problem of physiology; however, sleep appears to have a central role in his model, since dreaming is considered the guardian of sleep. The function of dreaming, according to Freud, is to protect sleep from disruption, with the dream working to avoid repressed stimuli interrupting the “biological” function of sleep. Before neurophysiological studies provided evidence that sleep is not a passive state, Freud also recognized sleep as an active process, as human beings voluntarily withdraw their attention from the external world to actively move to sleep. The discovery of REM sleep in the 1950s led psychoanalysts to see sleep as the necessary background to the occurrence of dreaming. Although Freud dismissed the clinical importance of sleep disturbances, viewing them as the somatic expression of an instinctual disturbance which would disappear during psychoanalytic treatment, successive authors highlighted the fact that sleep disturbances might have a more specific psychological significance. The similarities between the loss of self that occurs during sleep and the fragmentation of the identity experienced during schizophrenia represent an interesting and yet not fully explored area of research. Thanks to Freud’s work, the desire to sleep assumes the important role of a psychological, active factor that contributes to the occurrence and function of sleep.

## 1. Introduction

At the time when Freud published his seminal work on the interpretation of dreams [1], which would reveal the “unconscious” and its relationship with dreaming, scientific knowledge about sleep was limited and, as stated by Eisler in his influential paper “Pleasure in sleep and disturbed capacity for sleep” [2], published in 1922, “Science has so far treated the biological phenomenon of sleep descriptively, but it is unable to explain it satisfactorily as a dynamic process. Our assured knowledge regarding sleep is very deficient. We recognize it as a fundamental phenomenon in the organic world, which, like breathing and taking nourishment, aids in the periodic recuperation of the individual”.

It was only with the discovery of REM sleep by Aserinsky and Kleitman [3] about fifty years later, as well as the successive identification of slow-wave sleep (SWS) [4], also known as “delta sleep” [5], that a “Copernican revolution” brought a dramatic change in the neurophysiological and psychological field, leading to what is our current understanding of sleep and concomitant mental activity.

The two cited sleep components, SWS and REM sleep, have different functions and are controlled by different mechanisms [6,7], and they also tend to occur during different parts of the sleep period.

SWS occurs at the beginning of sleep and involves the progressive depletion of the cumulated sleep pressure [8]. REM sleep occurs cyclically and is expressed more at the end of the sleep period, and it is controlled by short- and long-term homeostatic regulations [9,10] as well as by the circadian clock [11,12].

Recent work has highlighted the role of these two components in memory consolidation: memory replay occurs during SWS in hippocampal assemblies, whereas REM sleep following SWS might balance local synaptic rescaling accompanying memory transformation [13].

As stated in *The Interpretation of Dreams*, Freud was not interested in sleep: “I have had little occasion to deal with the problem of sleep, for that is essentially a problem of physiology, even though one of the characteristics of the state of sleep must be that it brings about modifications of the conditions of functioning of the mental apparatus” [1].

However, sleep appears to have a fundamental role in his theory, since dreaming serves to protect sleep, and the dream work is to prevent interruptions to the “biological” function of sleep. Freud also underscores sleep as an active psychological state and as a reactivation of the sojourn in the womb.

In his “Introductory Lectures to Psychoanalysis” [14], Freud stated the following: “What, then, is sleep?” That is a physiological or biological problem concerning which much is still in dispute. We come to no decisive answer, but I think we may attempt to define one psychological characteristic of sleep. Sleep is a condition in which I refuse to have anything to do with the outer world and have withdrawn my interest from it. I go to sleep by retreating from the outside world and warding off the stimuli proceeding from it. Again, when I am tired of that world I go to sleep. I say to it as I fall asleep: “Leave me in peace for I want to sleep”.

According to Freud, human beings voluntarily withdraw their attention from the external world to actively move to sleep: “We are not in the habit of devoting much thought to the fact that every night human beings lay aside the wrappings in which they have enveloped their skin as well as anything they may use as a supplement to their bodily organ, for instance, their spectacles, their false hair and teeth and so on. We may add that when they go to sleep, they carry out an entirely analogous undressing of their minds and lay aside most of their psychical acquisitions” [15].

By characterizing sleep as a withdrawal from external reality, Freud established an important point, changing the naïve view of sleep as a passive condition to that of an active process. Thus, well before neurophysiology provided objective evidence of this, sleep and its dream function became a state of activity, also bringing a new perspective to the inner mental world: “It is, of course, the study of dreams which has taught us what we know of the mental characteristics of sleep. It is true that dreams only show us the dreamer in so far as he is not asleep; nevertheless, they are bound to reveal to us characteristics of sleep itself at the same time” [15].

This breakthrough in the view of sleep has been highlighted by Potzl [16]: “’Freud’s conception of sleep which has emerged from the purely psychic is fully in accord with the results of biological observation of the problem of sleep and with those modern theories of sleep which emphasize the active quality in sleep, the wish to withdraw; and yet the Freudian concept is older than those theories”.

Freud also suggested a similarity between sleep and prebirth life: “Sleep is somatically a re-activation of the sojourn in the womb, fulfilling the same conditions of restful posture, warmth and absence of stimuli; indeed, many people assume in sleep the fetal attitude. The psychic condition of a person asleep is characterized by an almost complete withdrawal from his environment and all interest in it. Thus, on both counts they approach remarkably close to the situation in which they began life” [15].

The regression to the prenatal state associated with the sleep state is further analyzed by Freud in his “Introductory Lectures to Psychoanalysis” [14]: “We can say in the light of the libido theory that sleep is a state in which all investments of objects, both libidinal and egoistic, are given up and withdrawn into the ego. Does this not throw a new light on recuperation by sleep and on the nature of fatigue? The picture of blissful isolation in intrauterine life, which the sleeping person conjures up again every night is, thus, confirmed and amplified on the mental side. In the sleeper the primal state of the libido-distribution is again reproduced, that of absolute narcissism, in which libido and ego-interests dwell together still, united and indistinguishable in the self-sufficient Self”.

The psychological function of sleep has been further discussed by Simmel in a contribution to the Symposium on Neurotic Disturbances of Sleep [17]: “During sleep, the ego has an opportunity of recovering from present and past injuries to its narcissism by nightly regression to earlier stages of instinctual development. The fact that these nightly regressions are associated with a temporary denial of reality on the one hand and with a blocking of the outward motor brain centers on the other gives sleep the character of a psycho-physiological method of defense against dangerous collisions between the ego and a surrounding world hostile to its instinctual demands”.

Considering that the biological function of sleep resides in the physiological restorative need, the concomitant psychological need is to withdraw from the real world, returning to a prenatal condition and moving into a state of primary narcissism.

Thanks to Freud’s work, the desire to sleep, therefore, assumes the important role of a psychological, active factor that contributes to the occurrence and function of sleep.

## 2. Dream: The Guardian of Sleep

### 2.1. Energy Reflux and Motor Inhibition

The biological need for sleep is the central element of Freud’s original dream theory.

The seventh chapter of *The Interpretation of Dreams* is in continuity with the “Project for a Scientific Psychology” [18]: “Energy”, which during wakefulness moves from perception, through memory, to motricity, cannot progress to motricity during sleep due to motor sleep paralysis; thus, it will go back, regressing to perception, reactivating a repressed desire in the unconscious and finally producing the dream, a hallucinatory satisfaction of a removed infant desire.

According to Freud, the dream is the night watch, the guardian of sleep. The function of dreaming is to protect sleep from disruption, and the dream work is to prevent repressed sexual stimuli from disturbing the course of sleep, interrupting its “biological” function. “There is only one useful task, only one function, that can be ascribed to a dream, and that is the guarding of sleep from interruption” [1].

### 2.2. Condensation, Displacement, and Representation of Principles of Dreams

The sleep condition, by reducing conscious censorship, allows sexual and aggressive drives, mixed up with memories and residue from the day, to be “represented” in dreams.

Dreams work by using the principles of condensation, displacement, and representation to transform unconscious conflicts, which are unacceptable to consciousness, into manifest accessible content. The dream is then an acceptable compromise between the primitive drive and the requirements of censorship, and the motor paralysis during sleep guarantees that the dreamer cannot act the dream.

“A dream tells us that something was going on which tended to interrupt sleep and enables us to understand in what way it has been possible to fend off the interruption. The outcome is that the sleeper has dreamt and is able to go on sleeping; the internal demand which was striving to occupy him has been replaced by an external experience whose demand has been disposed of. A dream is therefore amongst other things a projection; an externalization of an internal process” [1].

The act of dreaming provides the function of keeping sleep away from disturbing thoughts, as “dreaming dissipates the threat of overwhelming anxiety caused by the tension of repressed impulses originating in childhood” [19]. In Freud’s psychic energy model, “The dream safeguards sleep by acting as a safety valve, allowing sleep to continue by letting off just enough tension from unconscious impulses” [19].

### 2.3. Ego Vigilance and Sleep Depth

It is the pre-conscious that oversees the dream’s work of disguising unconscious wishes and annulling the disturbing effects of internal perception, but on the other hand, according to Simmel: “the individual will rarely achieve deepest level of sleep, because the ego is striving for hallucinatory wish-fulfilments derived from, or repressed from, later stages of libidinal development” [17]. A profound level of sleep, almost dreamless, can be achieved when the ego is not disturbed by the perception of stimuli from both the outside world and the inner psychical reality.

Sleeping drugs can influence difficulties in both falling asleep and deepening sleep, protecting the ego against stimuli from “without” or from “within”, respectively [17].

Freud also highlighted that the dreaming condition is associated with a sort of “awakening” of the sleeper—sleep is not considered as the opposite of wakefulness, but it is a complementary state to the waking state, and the two alternate with each other.

Jekels pointed out, following Freud, that “we know the dreamer as one who is awake in sleep, who is active in this phase of sleep and that the whole state of sleep is one of differing levels of activities, from active withdrawal to active awakening” [20].

Before electroencephalographic studies showed the alternating levels of consciousness during the night, psychoanalysts were, thus, already aware of a sleeping–waking cycle at night.

## 3. Sleep: The Guardian of Dreams

Since the discovery of REM sleep, the neurophysiological literature has associated dreaming with this sleep phase, leading to hypotheses that have attributed the origin of dreaming activity to the physiological and neurochemical activity occurring during REM sleep [21,22]. According to Hobson and McCarley [23,24], dreams are generated by the activation of neural activity in the brainstem and its signal transmission to the cortex. More recent work has also suggested dopamine activity in the forebrain as a possible mechanism of dream activity [25]. Evidence from neuroimaging studies [26,27] suggests that during REM sleep there is activation of the pons, the amygdala, and the anterior cingulate cortex, as well as deactivation of the posterior cingulate cortex and the dorsolateral prefrontal cortex, consistent with the emotionality and bizarreness of dream content.

Considering that mental activity is present during the whole sleep period, a useful categorization has distinguished mentation, which generally refers to thinking and is present throughout the whole sleep period, from dreaming, which refers to bizarre, disorganized mental activity. Dreams that occur during REM sleep tend to be longer, more vivid, more story-like, and more bizarre than those that occur during NREM sleep.

Thus, sleep, with its modification of the physiological condition and of the brain state, provides the necessary framework for the occurrence of the dreaming activity.

Pines has suggested that “the dictum that the dream is the guardian of sleep should now be reversed as EEG studies show that REM periods are the necessary background for dreaming; sleep, therefore, is the guardian of the dream” [28].

The close relationship between the occurrence of sleep and dreaming is well defined in Freud’s work: “Descriptive psychology tells us about the principal sine qua non for the formation of dreams is that the mind shall be in a state of sleep” […] “the state of sleep makes the formation of dreams possible because it reduces the power of the endo-psychic censorship” [1].

Jekels [20] underlines the relationship between dreams and sleep, also suggesting that dreaming occurs regularly every night: “The answer is, that except in the case of sudden awakening through an external stimulus, whenever there is sleep there is a dream. I am fully aware that in this I am not in agreement with most analysts and, above all, not with Freud. He held that the occurrence of a dream is conditioned by the day’s residues, the libidinal over cathexis of which is discharged by means of the dream. Although Freud considered the awakening function of the dream, he attached incomparably more significance to his role as a guardian of sleep which he had discovered”.

Furthermore, Jekels [20] attributes to dreaming the important role of preparing the ego for the process of awakening, considering, as also suggested by the works of Grotjahn [29] and Federn [30], the existence of a cognitive ego function “which never vanishes during sleep and remains extant to a certain degree even during deep sleep”. Interestingly, Jekels [20] also suggests that, as with sleep—which, due to Freud’s theory, is considered an active process—awakening should also be considered as an active process.

For psychoanalysis, sleep is, thus, a necessary condition for dreaming to occur. The motor paralysis that accompanies the sleep state prevents the dreamer from carrying out forbidden, dangerous actions. Sleep also helps to maintain the instinctual equilibrium of the ego, thanks to its organizing function, and it also allows dreaming to contain symbolization of sexual and aggressive fantasies.

## 4. Neurotic Disturbances of Sleep

One of sleep’s functions, as suggested by psychoanalytic studies, is to maintain the instinctual equilibrium of the ego and, through dreams, reduce disturbing sexual and aggressive drives. On the other hand, sleep can be interrupted by neurotic conflicts emerging from the unconscious, which induces anxiety, and that cannot be controlled by the censorship that works during wakefulness.

As stated by Fenichel in a seminal symposium dedicated to the “Neurotic disturbances of sleep”, “Anxiety, headaches, a state of depression and disturbances of sleep are among the most frequent complaints of patients seeing a psychiatrist” [31].

Fenichel also reflects that these alterations have been scarcely considered in psychoanalysis: But disturbances of sleep and headaches have found nowhere near so much attention among psychoanalysts. The reasons for this apparent lack of interest are to be found in Freud’s (1917) *Introductory Lectures* in the chapter dealing with Ordinary Nervousness: “These two types of symptoms are—or may be—of an actual neurotic nature, which explains why analysts who study the unconscious contents of symptoms have been less interested in them. Actual-neurotic symptoms are indicative of the fact that a disturbance in instinctual life is at work; but they do not reveal anything (or not enough) of the nature of the disturbance. Since they are a somatic expression of an instinctual disturbance, they disappear during psycho-analytic treatment, if the disturbance in question is of a psycho-genetic nature, without it being necessary to make them an explicit object of analytic attention. They disappear when the repression, which is the cause of the state of damming up and which is responsible for the symptom, is undone” [14].

Fenichel, however, suggests that not all sleep disturbances have a neurotic nature, and that in some cases they might have a specific psychological significance. “There are ‘sleep phobias’, in which the state of being asleep is more or less avoided because of a definite significance which that state has acquired in the unconscious mind of the patient; or there are ‘sleep rituals’ in which certain compulsive measures serve the purpose of putting out of action a definite danger which is unconsciously connected with the idea of being asleep: sleep becomes once more possible, in so far as this intention succeeds, whereas anxiety comes up again when the performance of the rituals is impeded” [31].

According to Fenichel, for an organism to fully benefit from the function of sleep, tension must be excluded from that organism. In neurotic disturbances of sleep, the difficulty in complete relaxation is determined by inner causes: “The same effect that an uncomfortable position or unconscious motor impulse can produce is even more frequently achieved by unconscious stimuli which are no longer under the control of the conscious wish to sleep and which still retain their cathexes” [31].

In contrast with Freud’s view, Fenichel considers impairment of the function of sleep as one of the most common neurotic manifestations, present in every neurosis. He also suggests that “such disturbances are relatively slight: some neurotics have learned to make the sleep-disturbing stimuli coming from the repressed harmless by applying secondary measures, by ‘canalizing’ them, as Windholz has shown, in various ways” [31].

“The fact that sometimes intense repressed cathexes seem not to interfere with the relaxation necessary for sleep obviously depends upon still other (constitutional?) factors, which we do not yet entirely understand. The question is analogous to that of dream frequency. As is well known, frequency of dreaming is by no means indicative of the quantity of the repressed. There are very normal people who dream nightly, and there are very neurotic people whose sleep is dreamless” [31].

Discussing sleep phobias, Fenichel suggests that “Fear of sleep means fear of the unconscious wishes that might arise in sleep. Frequently such a fear starts with an anxiety dream of traumatic effect. Fear of sleep is then a fear of dreaming as of an instinctual temptation” [31].

As an example of the fear of forbidden instinctual actions that assume the form of fear of sleep, Fenichel cites Berstein’s case of a child whose fear of falling asleep and dreaming was determined by their fear of losing control over their sphincters and soiling the bed during sleep. Similarly, in adults, fear of sleep is very often a fear of the temptation to masturbate. Also, Simmel [17], discussing patients with disturbances in their ability to fall asleep, suggests that during the initial stage of sleep, these patients show a tendency to postpone going to bed or falling asleep as a defense against masturbation, as experienced during childhood.

As pointed out by Fenichel, it should be kept in mind that the unconscious instinctual significance of the state of sleep cannot be applied schematically, since each case of sleep phobia should be analyzed singularly: “The unconscious meaning of ‘sleep’ that disturbs the function of sleep may be a specific and unique one which can only be explained by the life history of the individual” [31].

## 5. Sleep and Death

### 5.1. Death Instinct: From Mythological Metaphor to Biological Impulse

In *Beyond the Pleasure Principle* [32], Freud introduces an important element in his work—the death instinct, according to which all living multicellular species seek to return to a state of inorganic stability.

Sleep and death are closely linked in mankind’s imagination, as in several colloquial expressions we can find metaphors like “eternal sleep”, “to sleep like the dead”, and “death is a long sleep”. In Greek mythology, Thanatos, the god of death, and Hypnos, the god of sleep, are twins, sons of Night (Nyx) and Erebos. Morpheus, the god of dreams, is the son of Hypnos. Hermes is both the carrier of dreams and the bringer of death. The etymology of cemeteries is from “κοιμητήριον”, “coemeterium”: sleeping quarters. In Macbeth (Act 2, Scene2), “Sleep no more! Macbeth does murder sleep: the innocent sleep, Sleep that knits up the raveled sleeve of care, The death of each day’s life”.

### 5.2. Sleep Is the Cyclical Death of the Self

It should be considered that the sleep condition implies a loss of self and of the continuity of the ego, and the loss of consciousness can be associated with the idea of dying, whereas awakening is the restitution of the ego and the return to life. Kronfeld [33] believes that conditions like sleep and anxiety are a significant danger to the unity and wholeness of the person, implying an essential threat to the ego and an anticipation of death.

Federn [30], with his work on the ego in dreams, has highlighted the fact that “in full sleep ego feeling is extinguished”. The loss of the ego in sleep shares similarities with the fragmentation of the identity experienced during schizophrenia: “Just as the schizophrenic patient will feel that the world is dying when it is his own ego that is becoming threatened and weakened by withdrawal of energy or by eruption from the layers of the unconscious mind, so does sleep threaten us with deaths” [28].

### 5.3. Death Anxiety in Insomnia and Self-Dissolution in Schizophrenia

Both clinical researchers and psychoanalysts have associated sleep with schizophrenia. Schneider [34] found similarity between the disintegration of the ego occurring at sleep onset and that occurring during schizophrenia.

In his elegant paper “A bioanalytical contribution to the problem of sleep and wakefulness” [20], Jekels discusses the parallelism between sleep and schizophrenia: “In focusing attention on the sleeper’s withdrawal from reality, on his repudiation of the outside world, Freud has endowed him with the main characteristic of the schizophrenic, with his autism. In fact, the very foundation of Freud’s dream theory presupposes the disintegration of the ego, a disintegration which was considered the essential pathognomonic symptom of schizophrenia even before Bleuler (Wernicke’s dementia sejunctiva) and certainly since Bleuler”.

The periodic “wake” that occurs during dreams can serve to preserve the idea of life, helping the dreamer to “stay” alive, as originally suggested by Kant: “to excite internally the vital organs by the medium of the Imagination “…” without this internal power of motion and this fatiguing unrest, on account of which we complain about our dreams (though in fact they are rather remedial),sleep even in a sound state of health would be a complete extinction of life” [35].

According to Rotheberg [36], the fear of death associated with sleep can also lead to avoiding sleep, or to insomnia: “the deeper meaning of insomnia as a fear reaction to life situations which in the unconscious would stand for death, a condition to be avoided by the preconscious and by conscious wakefulness”.

## 6. Discussion

The role of the psychological factors of sleep, highlighted by Freud in *The Interpretation of Dreams*, is a fundamental and under-recognized revolution in the understanding of sleep functions. Sleep has not only become important for its restorative physiological attributes, but has also acquired a critical psychological significance.

The research on dreams, as well as the recognition of mental activity during sleep, has opened an entirely new field of investigation and knowledge about sleep. The successive work of Bion [37] has also extended Freud’s theory about dreaming to a process of emotional thinking that does not end with sleep, but continues during wakefulness, producing the necessary background to process emotional experiences.

Thanks to psychoanalytic studies, sleep can, thus, be viewed not as the opposite of wakefulness, but as a complement to the waking state.

It is worth noting that according to Freud’s original theory, the function of dreaming fundamentally serves to protect sleep from disruption, guaranteeing its biological need. This theory has recently been challenged by an interesting paper by Guenole et al. [38], who analyzed the available literature on the relationship between dreaming and arousal, with results from non-dreaming brain-damaged patients appearing to support Freud’s theory.

The wish to sleep, the voluntary withdrawal from the external world, from reality, that is expressed in Freud’s work has also changed the traditional view of sleep as a passive state to that of an active process, a concept that only became documented in the neurophysiological field through the work of Moruzzi and Magoun [39] in the late 1940s and the discovery of REM sleep [3] in the 1950s.

Although the protection of sleep appears to be the leading factor of his dream theory, Freud had no interest in sleep or its mechanisms, and considered sleep disturbances as secondary phenomena that psychotherapeutic interventions could fully resolve. However, as observed by authors like Fenichel, sleep disturbances may have a specific psychological significance that can be addressed and understood.

Whereas biological, cognitive, and behavioral approaches have been frequently used in the understanding and treatment of sleep disorders, psychoanalytic models have rarely been used. de Kernier et al. [40] have underscored the importance of intrapsychic conflicts in the emergence of sleep disorders during adolescence, and have also discussed the unconscious association between death and sleep. Schonning et al. [41] recently reported that symptoms of insomnia in adolescents with major depressive disorder improved following short-term psychoanalytic psychotherapy for depression. Psychoanalytic research on the ego, beginning with the work of Federn [30], has pointed out similarities between the loss of self which occurs by falling asleep and the fragmentation of the identity experienced during schizophrenia, which is an interesting and yet not fully explored area of research.

The psychological aspects of sleep remain a fertile area of research. There is still a lack of clarity regarding the functions of sleep and its components like REM sleep [42], which is the privileged dreaming phase that also appears to have a significant role in the dissolution of emotional distress [43,44].
